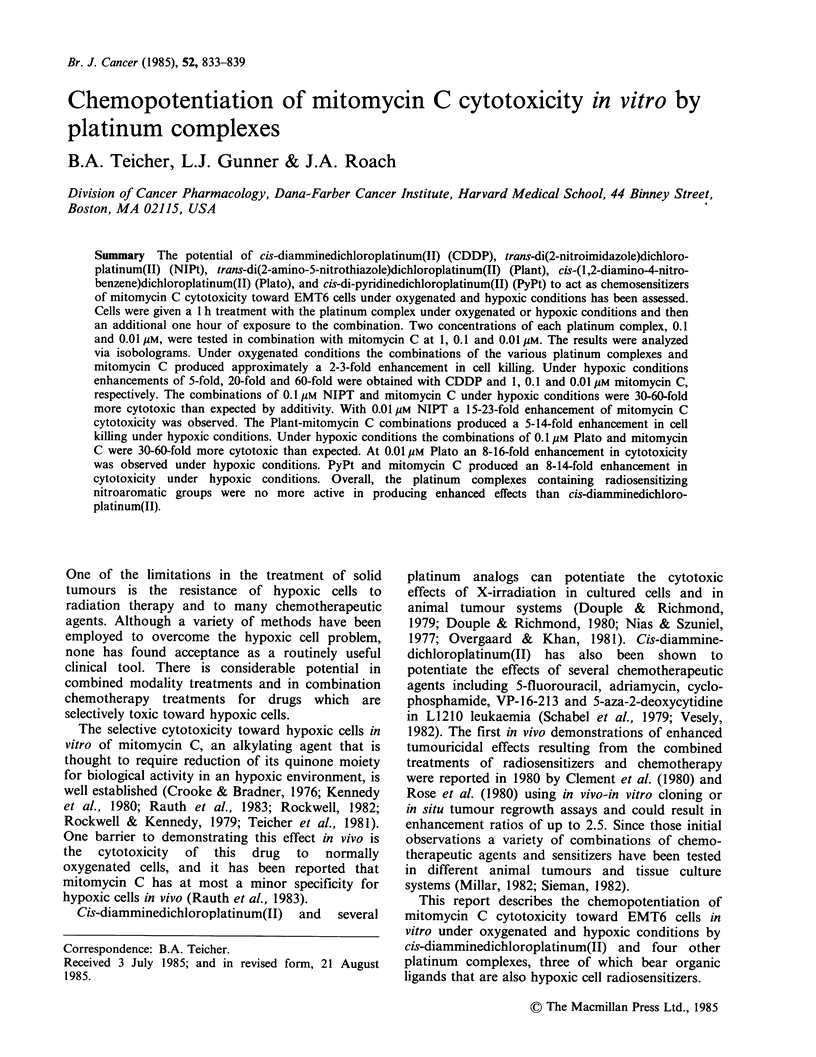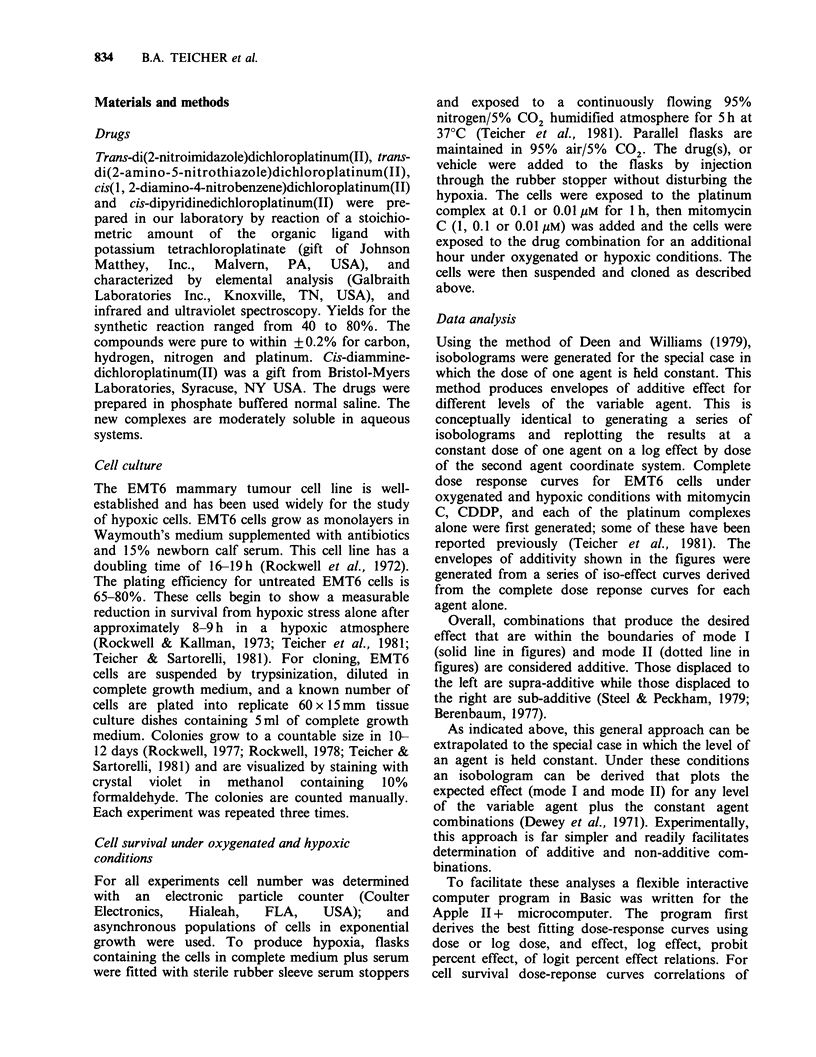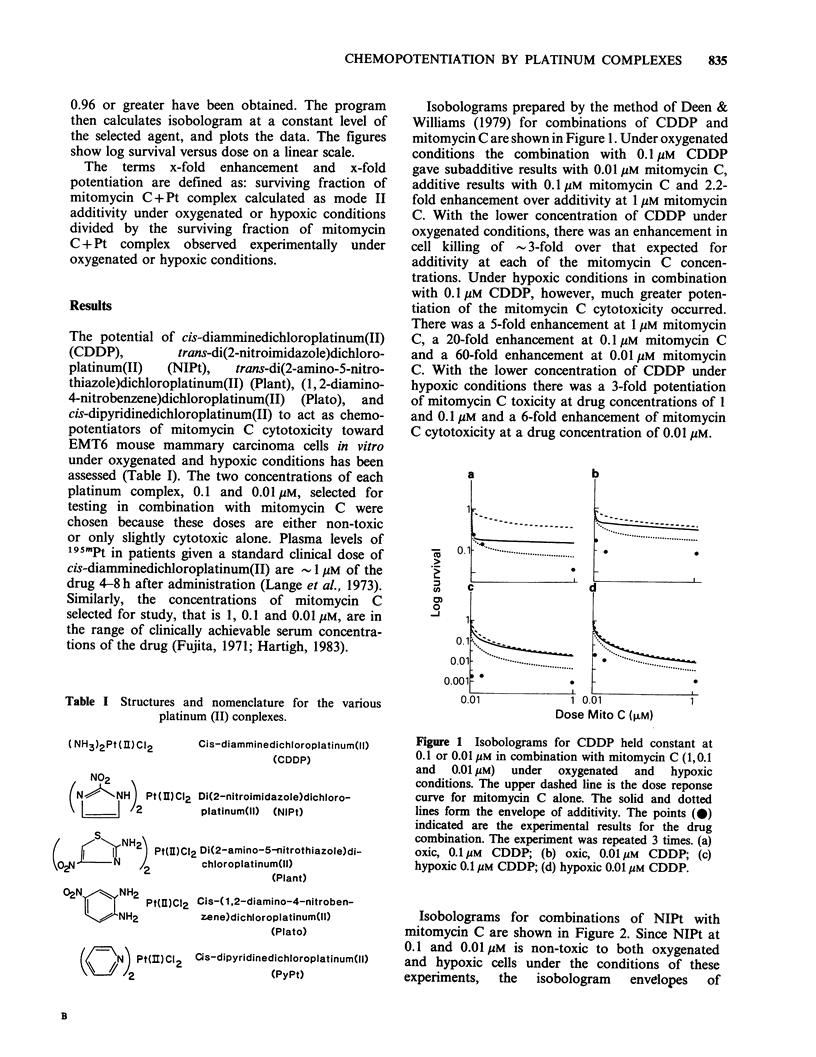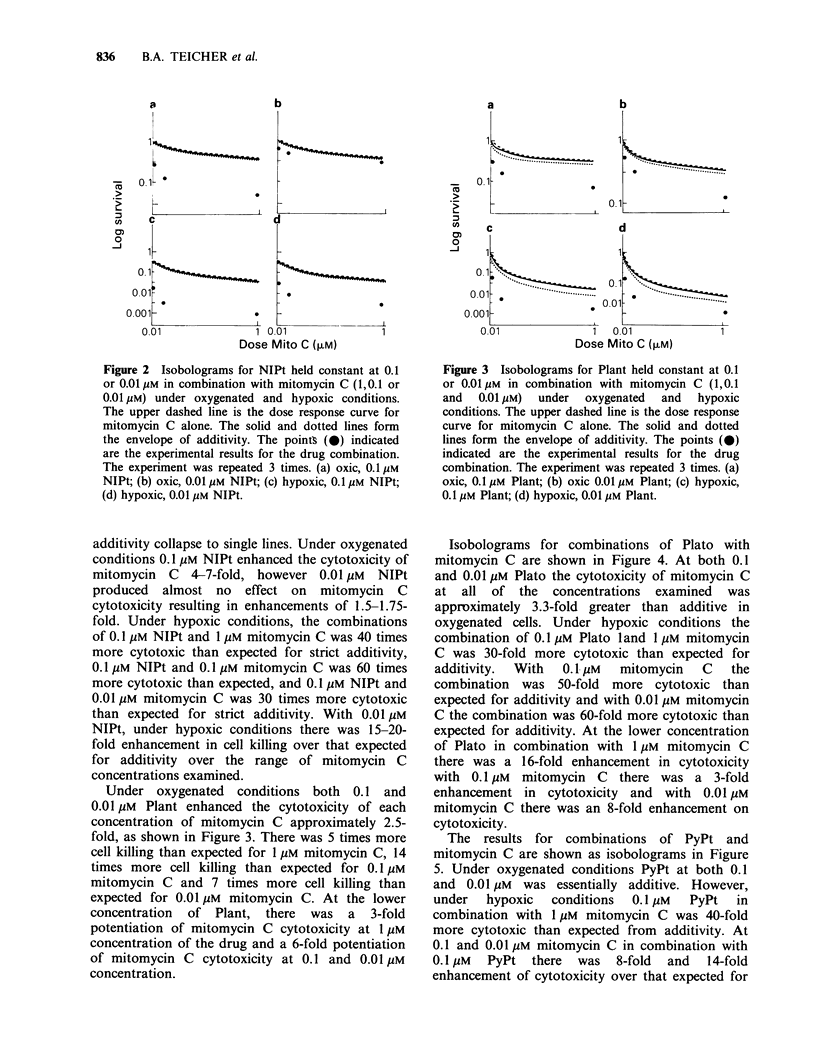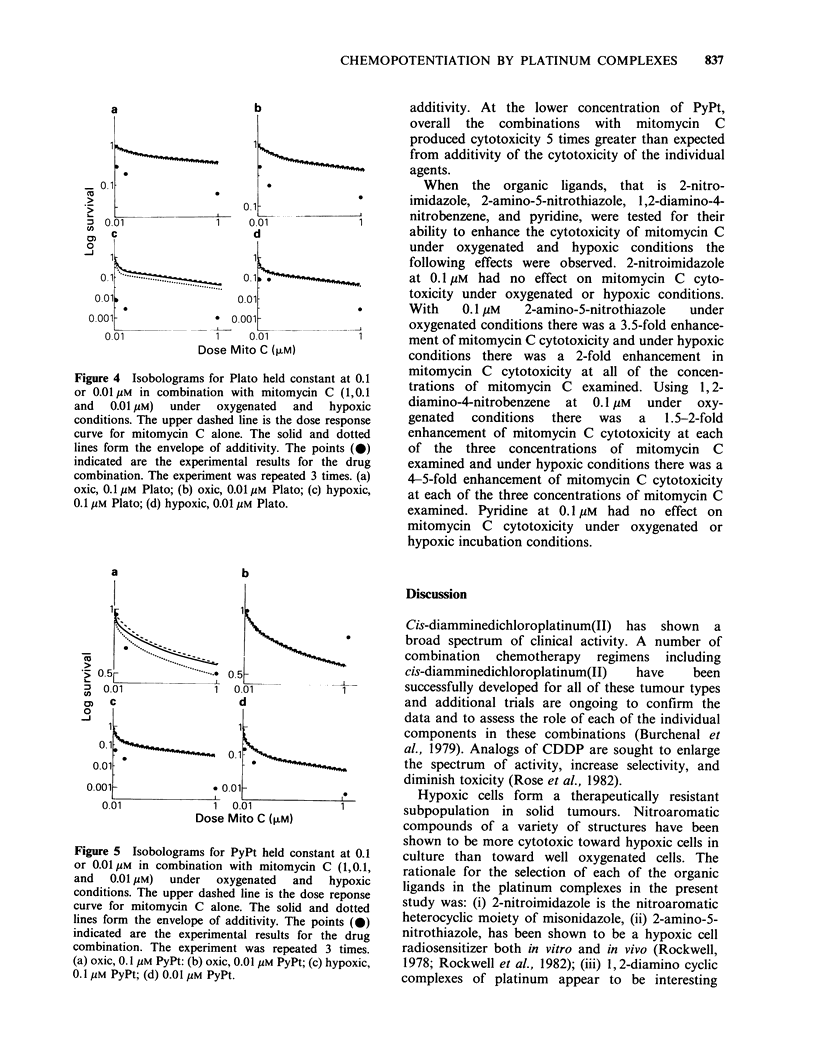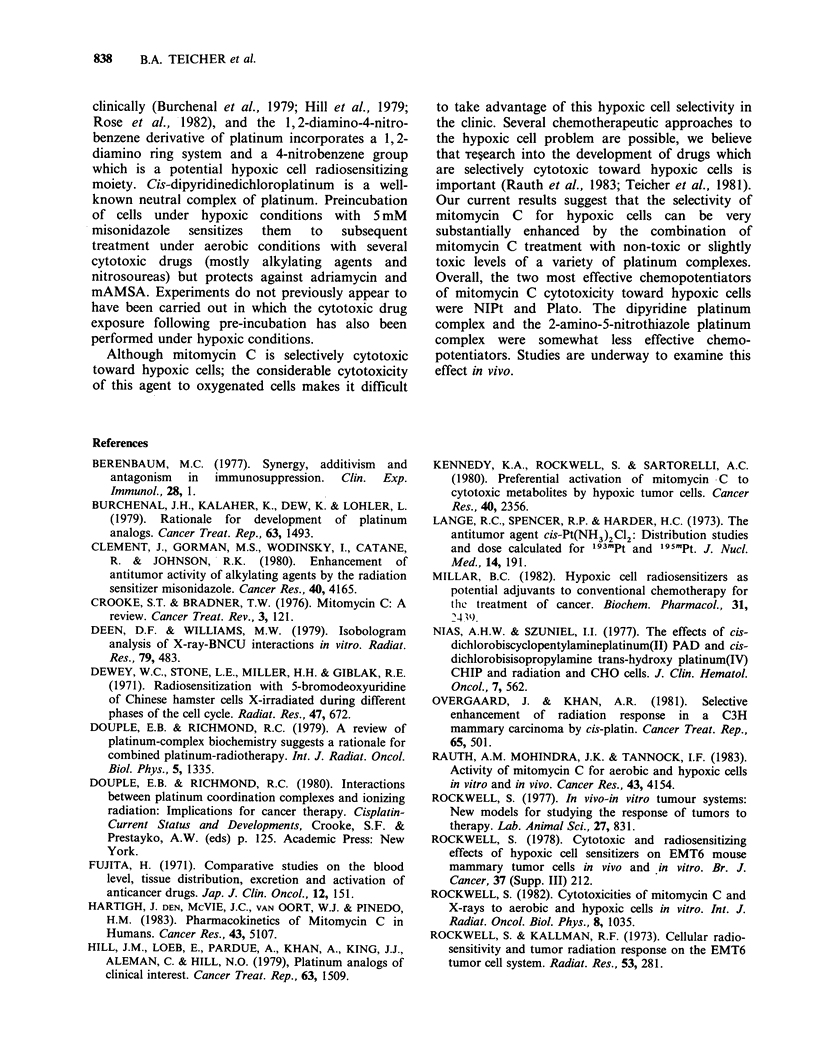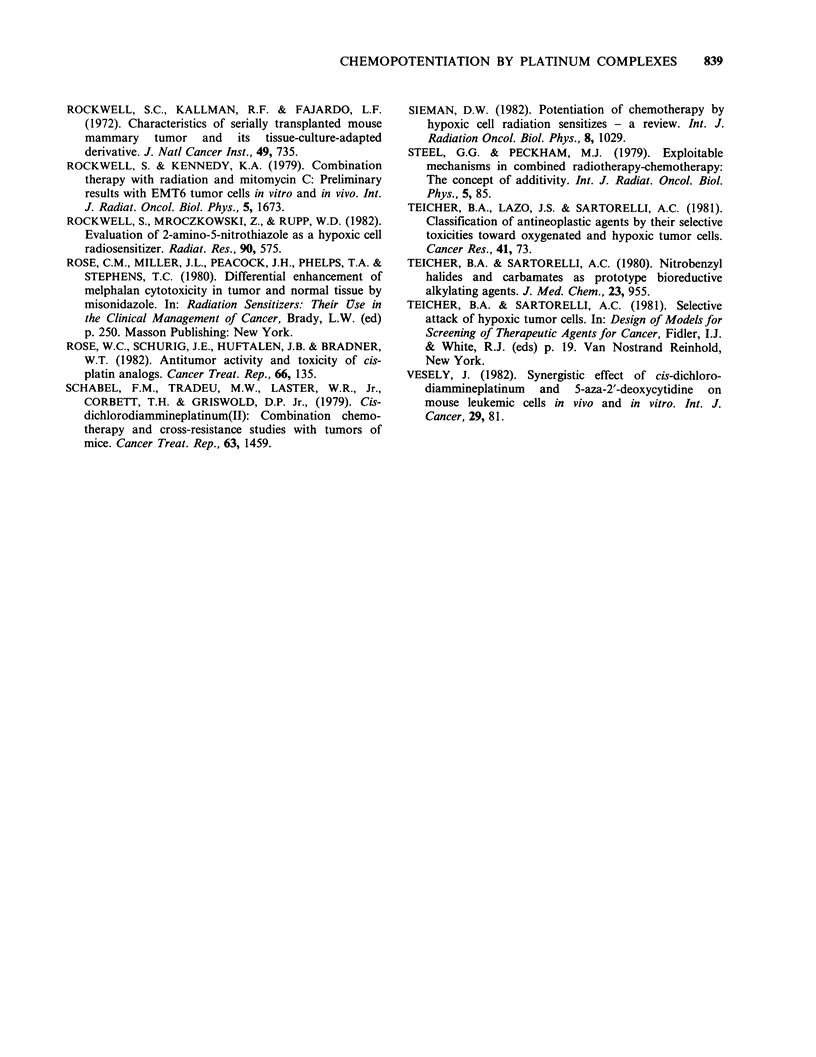# Chemopotentiation of mitomycin C cytotoxicity in vitro by platinum complexes.

**DOI:** 10.1038/bjc.1985.266

**Published:** 1985-12

**Authors:** B. A. Teicher, L. J. Gunner, J. A. Roach

## Abstract

The potential of cis-diamminedichloroplatinum(II) (CDDP), trans-di(2-nitroimidazole)dichloro-platinum(II) (NIPt), trans-di(2-amino-5-nitrothiazole)dichloroplatinum(II) (Plant), cis-(1,2-diamino-4-nitrobenzene)dichloroplatinum(II) (Plato), and cis-di-pyridinedichloroplatinum(II) (PyPt) to act as chemosensitizers of mitomycin C cytotoxicity toward EMT6 cells under oxygenated and hypoxic conditions has been assessed. Cells were given a 1 h treatment with the platinum complex under oxygenated or hypoxic conditions and then an additional one hour of exposure to the combination. Two concentrations of each platinum complex, 0.1 and 0.01 microM, were tested in combination with mitomycin C at 1, 0.1 and 0.01 microM. The results were analyzed via isobolograms. Under oxygenated conditions the combinations of the various platinum complexes and mitomycin C produced approximately a 2-3-fold enhancement in cell killing. Under hypoxic conditions enhancements of 5-fold, 20-fold and 60-fold were obtained with CDDP and 1, 0.1 and 0.01 microM mitomycin C, respectively. The combinations of 0.1 microM NIPT and mitomycin C under hypoxic conditions were 30-60-fold more cytotoxic than expected by additivity. With 0.01 microM NIPT a 15-23-fold enhancement of mitomycin C cytotoxicity was observed. The Plant-mitomycin C combinations produced a 5-14-fold enhancement in cell killing under hypoxic conditions. Under hypoxic conditions the combinations of 0.1 microM Plato and mitomycin C were 30-60-fold more cytotoxic than expected. At 0.01 microM Plato an 8-16-fold enhancement in cytotoxicity was observed under hypoxic conditions. PyPt and mitomycin C produced an 8-16-fold enhancement in cytotoxicity under hypoxic conditions. Overall, the platinum complexes containing radiosensitizing nitroaromatic groups were no more active in producing enhanced effects than cis-diamminedichloroplatinum(II).


*Br. J. Cancer* (1985), **52**, 833–839

### Chemopotentiation of mitomycin C cytotoxicity *in vitro* by platinum complexes

B.A. Teicher, L.J. Gunner & J.A. Roach

*Division of Cancer Pharmacology, Dana-Farber Cancer Institute, Harvard Medical School, 44 Binney Street, Boston, MA 02115, USA*

**Summary** The potential of *cis*-diamminedichloroplatinum(II) (CDDP), *trans*-di(2-nitroimidazole)dichloroplatinum(II) (NIPt), *trans*-di(2-amino-5-nitrothiazole)dichloroplatinum(II) (Plant), *cis*-(1,2-diamino-4-nitrobenzene)dichloroplatinum(II) (Plato), and *cis*-di-pyridinedichloroplatinum(II) (PyPt) to act as chemosensitizers of mitomycin C cytotoxicity toward EMT6 cells under oxygenated and hypoxic conditions has been assessed. Cells were given a 1 h treatment with the platinum complex under oxygenated or hypoxic conditions and then an additional one hour of exposure to the combination. Two concentrations of each platinum complex, 0.1 and 0.01 $\mu$M, were tested in combination with mitomycin C at 1, 0.1 and 0.01 $\mu$M. The results were analyzed via isobolograms. Under oxygenated conditions the combinations of the various platinum complexes and mitomycin C produced approximately a 2-3-fold enhancement in cell killing. Under hypoxic conditions enhancements of 5-fold, 20-fold and 60-fold were obtained with CDDP and 1, 0.1 and 0.01 $\mu$M mitomycin C, respectively. The combinations of 0.1 $\mu$M NIPT and mitomycin C under hypoxic conditions were 30-60-fold more cytotoxic than expected by additivity. With 0.01 $\mu$M NIPT a 15-23-fold enhancement of mitomycin C cytotoxicity was observed. The Plant-mitomycin C combinations produced a 5-14-fold enhancement in cell killing under hypoxic conditions. Under hypoxic conditions the combinations of 0.1 $\mu$M Plato and mitomycin C were 30-60-fold more cytotoxic than expected. At 0.01 $\mu$M Plato an 8-16-fold enhancement in cytotoxicity was observed under hypoxic conditions. PyPt and mitomycin C produced an 8-14-fold enhancement in cytotoxicity under hypoxic conditions. Overall, the platinum complexes containing radiosensitizing nitroaromatic groups were no more active in producing enhanced effects than *cis*-diamminedichloroplatinum(II).

One of the limitations in the treatment of solid tumours is the resistance of hypoxic cells to radiation therapy and to many chemotherapeutic agents. Although a variety of methods have been employed to overcome the hypoxic cell problem, none has found acceptance as a routinely useful clinical tool. There is considerable potential in combined modality treatments and in combination chemotherapy treatments for drugs which are selectively toxic toward hypoxic cells.

The selective cytotoxicity toward hypoxic cells *in vitro* of mitomycin C, an alkylating agent that is thought to require reduction of its quinone moiety for biological activity in an hypoxic environment, is well established (Crooke & Bradner, 1976; Kennedy *et al.*, 1980; Rauth *et al.*, 1983; Rockwell, 1982; Rockwell & Kennedy, 1979; Teicher *et al.*, 1981). One barrier to demonstrating this effect *in vivo* is the cytotoxicity of this drug to normally oxygenated cells, and it has been reported that mitomycin C has at most a minor specificity for hypoxic cells *in vivo* (Rauth *et al.*, 1983).

*Cis*-diamminedichloroplatinum(II) and several platinum analogs can potentiate the cytotoxic effects of X-irradiation in cultured cells and in animal tumour systems (Douple & Richmond, 1979; Douple & Richmond, 1980; Nias & Szuniel, 1977; Overgaard & Khan, 1981). *Cis*-diamminedichloroplatinum(II) has also been shown to potentiate the effects of several chemotherapeutic agents including 5-fluorouracil, adriamycin, cyclophosphamide, VP-16-213 and 5-aza-2-deoxycytidine in L1210 leukaemia (Schabel *et al.*, 1979; Vesely, 1982). The first *in vivo* demonstrations of enhanced tumouricidal effects resulting from the combined treatments of radiosensitizers and chemotherapy were reported in 1980 by Clement *et al.* (1980) and Rose *et al.* (1980) using *in vivo-in vitro* cloning or *in situ* tumour regrowth assays and could result in enhancement ratios of up to 2.5. Since those initial observations a variety of combinations of chemotherapeutic agents and sensitizers have been tested in different animal tumours and tissue culture systems (Millar, 1982; Sieman, 1982).

This report describes the chemopotentiation of mitomycin C cytotoxicity toward EMT6 cells *in vitro* under oxygenated and hypoxic conditions by *cis*-diamminedichloroplatinum(II) and four other platinum complexes, three of which bear organic ligands that are also hypoxic cell radiosensitizers.

Correspondence: B.A. Teicher.
Received 3 July 1985; and in revised form, 21 August 1985.

© The Macmillan Press Ltd., 1985

### Materials and methods

### *Drugs*

*Trans*-di(2-nitroimidazole)dichloroplatinum(II), *trans*-di(2-amino-5-nitrothiazole)dichloroplatinum(II), *cis*(1, 2-diamino-4-nitrobenzene)dichloroplatinum(II) and *cis*-dipyridinedichloroplatinum(II) were prepared in our laboratory by reaction of a stoichiometric amount of the organic ligand with potassium tetrachloroplatinate (gift of Johnson Matthey, Inc., Malvern, PA, USA), and characterized by elemental analysis (Galbraith Laboratories Inc., Knoxville, TN, USA), and infrared and ultraviolet spectroscopy. Yields for the synthetic reaction ranged from 40 to 80%. The compounds were pure to within $\pm 0.2\%$ for carbon, hydrogen, nitrogen and platinum. *Cis*-diamminedichloroplatinum(II) was a gift from Bristol-Myers Laboratories, Syracuse, NY USA. The drugs were prepared in phosphate buffered normal saline. The new complexes are moderately soluble in aqueous systems.

### *Cell culture*

The EMT6 mammary tumour cell line is well-established and has been used widely for the study of hypoxic cells. EMT6 cells grow as monolayers in Waymouth's medium supplemented with antibiotics and 15% newborn calf serum. This cell line has a doubling time of 16–19 h (Rockwell *et al.*, 1972). The plating efficiency for untreated EMT6 cells is 65–80%. These cells begin to show a measurable reduction in survival from hypoxic stress alone after approximately 8–9 h in a hypoxic atmosphere (Rockwell & Kallman, 1973; Teicher *et al.*, 1981; Teicher & Sartorelli, 1981). For cloning, EMT6 cells are suspended by trypsinization, diluted in complete growth medium, and a known number of cells are plated into replicate 60 × 15 mm tissue culture dishes containing 5 ml of complete growth medium. Colonies grow to a countable size in 10–12 days (Rockwell, 1977; Rockwell, 1978; Teicher & Sartorelli, 1981) and are visualized by staining with crystal violet in methanol containing 10% formaldehyde. The colonies are counted manually. Each experiment was repeated three times.

### *Cell survival under oxygenated and hypoxic conditions*

For all experiments cell number was determined with an electronic particle counter (Coulter Electronics, Hialeah, FLA, USA); and asynchronous populations of cells in exponential growth were used. To produce hypoxia, flasks containing the cells in complete medium plus serum were fitted with sterile rubber sleeve serum stoppers and exposed to a continuously flowing 95% nitrogen/5% $CO_2$ humidified atmosphere for 5 h at 37°C (Teicher *et al.*, 1981). Parallel flasks are maintained in 95% air/5% $CO_2$. The drug(s), or vehicle were added to the flasks by injection through the rubber stopper without disturbing the hypoxia. The cells were exposed to the platinum complex at 0.1 or 0.01 $\mu$M for 1 h, then mitomycin C (1, 0.1 or 0.01 $\mu$M) was added and the cells were exposed to the drug combination for an additional hour under oxygenated or hypoxic conditions. The cells were then suspended and cloned as described above.

### *Data analysis*

Using the method of Deen and Williams (1979), isobolograms were generated for the special case in which the dose of one agent is held constant. This method produces envelopes of additive effect for different levels of the variable agent. This is conceptually identical to generating a series of isobolograms and replotting the results at a constant dose of one agent on a log effect by dose of the second agent coordinate system. Complete dose response curves for EMT6 cells under oxygenated and hypoxic conditions with mitomycin C, CDDP, and each of the platinum complexes alone were first generated; some of these have been reported previously (Teicher *et al.*, 1981). The envelopes of additivity shown in the figures were generated from a series of iso-effect curves derived from the complete dose reponse curves for each agent alone.

Overall, combinations that produce the desired effect that are within the boundaries of mode I (solid line in figures) and mode II (dotted line in figures) are considered additive. Those displaced to the left are supra-additive while those displaced to the right are sub-additive (Steel & Peckham, 1979; Berenbaum, 1977).

As indicated above, this general approach can be extrapolated to the special case in which the level of an agent is held constant. Under these conditions an isobologram can be derived that plots the expected effect (mode I and mode II) for any level of the variable agent plus the constant agent combinations (Dewey *et al.*, 1971). Experimentally, this approach is far simpler and readily facilitates determination of additive and non-additive combinations.

To facilitate these analyses a flexible interactive computer program in Basic was written for the Apple II+ microcomputer. The program first derives the best fitting dose-response curves using dose or log dose, and effect, log effect, probit percent effect, of logit percent effect relations. For cell survival dose-reponse curves correlations of

0.96 or greater have been obtained. The program then calculates isobologram at a constant level of the selected agent, and plots the data. The figures show log survival versus dose on a linear scale.

The terms x-fold enhancement and x-fold potentiation are defined as: surviving fraction of mitomycin C+Pt complex calculated as mode II additivity under oxygenated or hypoxic conditions divided by the surviving fraction of mitomycin C+Pt complex observed experimentally under oxygenated or hypoxic conditions.

### Results

The potential of *cis*-diamminedichloroplatinum(II) (CDDP), *trans*-di(2-nitroimidazole)dichloroplatinum(II) (NIPt), *trans*-di(2-amino-5-nitrothiazole)dichloroplatinum(II) (Plant), (1,2-diamino-4-nitrobenzene)dichloroplatinum(II) (Plato), and *cis*-dipyridinedichloroplatinum(II) to act as chemopotentiators of mitomycin C cytotoxicity toward EMT6 mouse mammary carcinoma cells *in vitro* under oxygenated and hypoxic conditions has been assessed (Table I). The two concentrations of each platinum complex, 0.1 and 0.01 µM, selected for testing in combination with mitomycin C were chosen because these doses are either non-toxic or only slightly cytotoxic alone. Plasma levels of $^{195m}$Pt in patients given a standard clinical dose of *cis*-diamminedichloroplatinum(II) are ~1 µM of the drug 4–8 h after administration (Lange *et al.*, 1973). Similarly, the concentrations of mitomycin C selected for study, that is 1, 0.1 and 0.01 µM, are in the range of clinically achievable serum concentrations of the drug (Fujita, 1971; Hartigh, 1983).

**Table I** Structures and nomenclature for the various platinum (II) conplexes.

| | |
|---|---|
| $(NH_3)_2Pt(II)Cl_2$ | Cis-diamminedichloroplatinum(II) (CDDP) |
|  Pt(II)Cl2 | Di(2-nitroimidazole)dichloroplatinum(II) (NIPt) |
|  Pt(II)Cl2 | Di(2-amino-5-nitrothiazole)dichloroplatinum(II) (Plant) |
|  Pt(II)Cl2 | Cis-(1,2-diamino-4-nitrobenzene)dichloroplatinum(II) (Plato) |
|  Pt(II)Cl2 | Cis-dipyridinedichloroplatinum(II) (PyPt) |

Isobolograms prepared by the method of Deen & Williams (1979) for combinations of CDDP and mitomycin C are shown in Figure 1. Under oxygenated conditions the combination with 0.1 µM CDDP gave subadditive results with 0.01 µM mitomycin C, additive results with 0.1 µM mitomycin C and 2.2-fold enhancement over additivity at 1 µM mitomycin C. With the lower concentration of CDDP under oxygenated conditions, there was an enhancement in cell killing of ~3-fold over that expected for additivity at each of the mitomycin C concentrations. Under hypoxic conditions in combination with 0.1 µM CDDP, however, much greater potentiation of the mitomycin C cytotoxicity occurred. There was a 5-fold enhancement at 1 µM mitomycin C, a 20-fold enhancement at 0.1 µM mitomycin C and a 60-fold enhancement at 0.01 µM mitomycin C. With the lower concentration of CDDP under hypoxic conditions there was a 3-fold potentiation of mitomycin C toxicity at drug concentrations of 1 and 0.1 µM and a 6-fold enhancement of mitomycin C cytotoxicity at a drug concentration of 0.01 µM.

**Figure 1** Isobolograms for CDDP held constant at 0.1 or 0.01 µM in combination with mitomycin C (1, 0.1 and 0.01 µM) under oxygenated and hypoxic conditions. The upper dashed line is the dose reponse curve for mitomycin C alone. The solid and dotted lines form the envelope of additivity. The points (●) indicated are the experimental results for the drug combination. The experiment was repeated 3 times. (a) oxic, 0.1 µM CDDP; (b) oxic, 0.01 µM CDDP; (c) hypoxic 0.1 µM CDDP; (d) hypoxic 0.01 µM CDDP.

Isobolograms for combinations of NIPt with mitomycin C are shown in Figure 2. Since NIPt at 0.1 and 0.01 µM is non-toxic to both oxygenated and hypoxic cells under the conditions of these experiments, the isobologram envelopes of

**Figure 2** Isobolograms for NIPt held constant at 0.1 or 0.01 μM in combination with mitomycin C (1, 0.1 or 0.01 μM) under oxygenated and hypoxic conditions. The upper dashed line is the dose response curve for mitomycin C alone. The solid and dotted lines form the envelope of additivity. The points (●) indicated are the experimental results for the drug combination. The experiment was repeated 3 times. (a) oxic, 0.1 μM NIPt; (b) oxic, 0.01 μM NIPt; (c) hypoxic, 0.1 μM NIPt; (d) hypoxic, 0.01 μM NIPt.

**Figure 3** Isobolograms for Plant held constant at 0.1 or 0.01 μM in combination with mitomycin C (1, 0.1 and 0.01 μM) under oxygenated and hypoxic conditions. The upper dashed line is the dose response curve for mitomycin C alone. The solid and dotted lines form the envelope of additivity. The points (●) indicated are the experimental results for the drug combination. The experiment was repeated 3 times. (a) oxic, 0.1 μM Plant; (b) oxic 0.01 μM Plant; (c) hypoxic, 0.1 μM Plant; (d) hypoxic, 0.01 μM Plant.

additivity collapse to single lines. Under oxygenated conditions 0.1 μM NIPt enhanced the cytotoxicity of mitomycin C 4–7-fold, however 0.01 μM NIPt produced almost no effect on mitomycin C cytotoxicity resulting in enhancements of 1.5–1.75-fold. Under hypoxic conditions, the combinations of 0.1 μM NIPt and 1 μM mitomycin C was 40 times more cytotoxic than expected for strict additivity, 0.1 μM NIPt and 0.1 μM mitomycin C was 60 times more cytotoxic than expected, and 0.1 μM NIPt and 0.01 μM mitomycin C was 30 times more cytotoxic than expected for strict additivity. With 0.01 μM NIPt, under hypoxic conditions there was 15–20-fold enhancement in cell killing over that expected for additivity over the range of mitomycin C concentrations examined.

Under oxygenated conditions both 0.1 and 0.01 μM Plant enhanced the cytotoxicity of each concentration of mitomycin C approximately 2.5-fold, as shown in Figure 3. There was 5 times more cell killing than expected for 1 μM mitomycin C, 14 times more cell killing than expected for 0.1 μM mitomycin C and 7 times more cell killing than expected for 0.01 μM mitomycin C. At the lower concentration of Plant, there was a 3-fold potentiation of mitomycin C cytotoxicity at 1 μM concentration of the drug and a 6-fold potentiation of mitomycin C cytotoxicity at 0.1 and 0.01 μM concentration.

Isobolograms for combinations of Plato with mitomycin C are shown in Figure 4. At both 0.1 and 0.01 μM Plato the cytotoxicity of mitomycin C at all of the concentrations examined was approximately 3.3-fold greater than additive in oxygenated cells. Under hypoxic conditions the combination of 0.1 μM Plato 1and 1 μM mitomycin C was 30-fold more cytotoxic than expected for additivity. With 0.1 μM mitomycin C the combination was 50-fold more cytotoxic than expected for additivity and with 0.01 μM mitomycin C the combination was 60-fold more cytotoxic than expected for additivity. At the lower concentration of Plato in combination with 1 μM mitomycin C there was a 16-fold enhancement in cytotoxicity with 0.1 μM mitomycin C there was a 3-fold enhancement in cytotoxicity and with 0.01 μM mitomycin C there was an 8-fold enhancement on cytotoxicity.

The results for combinations of PyPt and mitomycin C are shown as isobolograms in Figure 5. Under oxygenated conditions PyPt at both 0.1 and 0.01 μM was essentially additive. However, under hypoxic conditions 0.1 μM PyPt in combination with 1 μM mitomycin C was 40-fold more cytotoxic than expected from additivity. At 0.1 and 0.01 μM mitomycin C in combination with 0.1 μM PyPt there was 8-fold and 14-fold enhancement of cytotoxicity over that expected for

**Figure 4** Isobolograms for Plato held constant at 0.1 or 0.01 μM in combination with mitomycin C (1,0.1 and 0.01 μM) under oxygenated and hypoxic conditions. The upper dashed line is the dose response curve for mitomycin C alone. The solid and dotted lines form the envelope of additivity. The points (●) indicated are the experimental results for the drug combination. The experiment was repeated 3 times. (a) oxic, 0.1 μM Plato; (b) oxic, 0.01 μM Plato; (c) hypoxic, 0.1 μM Plato; (d) hypoxic, 0.01 μM Plato.

**Figure 5** Isobolograms for PyPt held constant at 0.1 or 0.01 μM in combination with mitomycin C (1,0.1, and 0.01 μM) under oxygenated and hypoxic conditions. The upper dashed line is the dose reponse curve for mitomycin C alone. The solid and dotted lines form the envelope of additivity. The points (●) indicated are the experimental results for the drug combination. The experiment was repeated 3 times. (a) oxic, 0.1 μM PyPt: (b) oxic, 0.01 μM PyPt; (c) hypoxic, 0.1 μM PyPt; (d) 0.01 μM PyPt.

additivity. At the lower concentration of PyPt, overall the combinations with mitomycin C produced cytotoxicity 5 times greater than expected from additivity of the cytotoxicity of the individual agents.

When the organic ligands, that is 2-nitroimidazole, 2-amino-5-nitrothiazole, 1,2-diamino-4-nitrobenzene, and pyridine, were tested for their ability to enhance the cytotoxicity of mitomycin C under oxygenated and hypoxic conditions the following effects were observed. 2-nitroimidazole at 0.1 μM had no effect on mitomycin C cytotoxicity under oxygenated or hypoxic conditions. With 0.1 μM 2-amino-5-nitrothiazole under oxygenated conditions there was a 3.5-fold enhancement of mitomycin C cytotoxicity and under hypoxic conditions there was a 2-fold enhancement in mitomycin C cytotoxicity at all of the concentrations of mitomycin C examined. Using 1,2-diamino-4-nitrobenzene at 0.1 μM under oxygenated conditions there was a 1.5–2-fold enhancement of mitomycin C cytotoxicity at each of the three concentrations of mitomycin C examined and under hypoxic conditions there was a 4–5-fold enhancement of mitomycin C cytotoxicity at each of the three concentrations of mitomycin C examined. Pyridine at 0.1 μM had no effect on mitomycin C cytotoxicity under oxygenated or hypoxic incubation conditions.

### Discussion

*Cis*-diamminedichloroplatinum(II) has shown a broad spectrum of clinical activity. A number of combination chemotherapy regimens including *cis*-diamminedichloroplatinum(II) have been successfully developed for all of these tumour types and additional trials are ongoing to confirm the data and to assess the role of each of the individual components in these combinations (Burchenal *et al.*, 1979). Analogs of CDDP are sought to enlarge the spectrum of activity, increase selectivity, and diminish toxicity (Rose *et al.*, 1982).

Hypoxic cells form a therapeutically resistant subpopulation in solid tumours. Nitroaromatic compounds of a variety of structures have been shown to be more cytotoxic toward hypoxic cells in culture than toward well oxygenated cells. The rationale for the selection of each of the organic ligands in the platinum complexes in the present study was: (i) 2-nitroimidazole is the nitroaromatic heterocyclic moiety of misonidazole, (ii) 2-amino-5-nitrothiazole, has been shown to be a hypoxic cell radiosensitizer both *in vitro* and *in vivo* (Rockwell, 1978; Rockwell *et al.*, 1982); (iii) 1,2-diamino cyclic complexes of platinum appear to be interesting clinically (Burchenal et al., 1979; Hill et al., 1979; Rose et al., 1982), and the 1,2-diamino-4-nitrobenzene derivative of platinum incorporates a 1,2-diamino ring system and a 4-nitrobenzene group which is a potential hypoxic cell radiosensitizing moiety. *Cis*-dipyridinedichloroplatinum is a well-known neutral complex of platinum. Preincubation of cells under hypoxic conditions with 5 mM misonidazole sensitizes them to subsequent treatment under aerobic conditions with several cytotoxic drugs (mostly alkylating agents and nitrosoureas) but protects against adriamycin and mAMSA. Experiments do not previously appear to have been carried out in which the cytotoxic drug exposure following pre-incubation has also been performed under hypoxic conditions.

Although mitomycin C is selectively cytotoxic toward hypoxic cells; the considerable cytotoxicity of this agent to oxygenated cells makes it difficult to take advantage of this hypoxic cell selectivity in the clinic. Several chemotherapeutic approaches to the hypoxic cell problem are possible, we believe that research into the development of drugs which are selectively cytotoxic toward hypoxic cells is important (Rauth et al., 1983; Teicher et al., 1981). Our current results suggest that the selectivity of mitomycin C for hypoxic cells can be very substantially enhanced by the combination of mitomycin C treatment with non-toxic or slightly toxic levels of a variety of platinum complexes. Overall, the two most effective chemopotentiators of mitomycin C cytotoxicity toward hypoxic cells were NIPt and Plato. The dipyridine platinum complex and the 2-amino-5-nitrothiazole platinum complex were somewhat less effective chemopotentiators. Studies are underway to examine this effect *in vivo*.

### References

BERENBAUM, M.C. (1977). Synergy, additivism and antagonism in immunosuppression. *Clin. Exp. Immunol.*, **28**, 1.

BURCHENAL, J.H., KALAHER, K., DEW, K. & LOHLER, L. (1979). Rationale for development of platinum analogs. *Cancer Treat. Rep.*, **63**, 1493.

CLEMENT, J., GORMAN, M.S., WODINSKY, I., CATANE, R. & JOHNSON, R.K. (1980). Enhancement of antitumor activity of alkylating agents by the radiation sensitizer misonidazole. *Cancer Res.*, **40**, 4165.

CROOKE, S.T. & BRADNER, T.W. (1976). Mitomycin C: A review. *Cancer Treat. Rev.*, **3**, 121.

DEEN, D.F. & WILLIAMS, M.W. (1979). Isobologram analysis of X-ray-BNCU interactions *in vitro*. *Radiat. Res.*, **79**, 483.

DEWEY, W.C., STONE, L.E., MILLER, H.H. & GIBLAK, R.E. (1971). Radiosensitization with 5-bromodeoxyuridine of Chinese hamster cells X-irradiated during different phases of the cell cycle. *Radiat. Res.*, **47**, 672.

DOUPLE, E.B. & RICHMOND, R.C. (1979). A review of platinum-complex biochemistry suggests a rationale for combined platinum-radiotherapy. *Int. J. Radiat. Oncol. Biol. Phys.*, **5**, 1335.

DOUPLE, E.B. & RICHMOND, R.C. (1980). Interactions between platinum coordination complexes and ionizing radiation: Implications for cancer therapy. *Cisplatin-Current Status and Developments*, Crooke, S.F. & Prestayko, A.W. (eds) p. 125. Academic Press: New York.

FUJITA, H. (1971). Comparative studies on the blood level, tissue distribution, excretion and activation of anticancer drugs. *Jap. J. Clin. Oncol.*, **12**, 151.

HARTIGH, J. DEN, MCVIE, J.C., VAN OORT, W.J. & PINEDO, H.M. (1983). Pharmacokinetics of Mitomycin C in Humans. *Cancer Res.*, **43**, 5107.

HILL, J.M., LOEB, E., PARDUE, A., KHAN, A., KING, J.J., ALEMAN, C. & HILL, N.O. (1979), Platinum analogs of clinical interest. *Cancer Treat. Rep.*, **63**, 1509.

KENNEDY, K.A., ROCKWELL, S. & SARTORELLI, A.C. (1980). Preferential activation of mitomycin C to cytotoxic metabolites by hypoxic tumor cells. *Cancer Res.*, **40**, 2356.

LANGE, R.C., SPENCER, R.P. & HARDER, H.C. (1973). The antitumor agent *cis*-$Pt(NH_3)_2Cl_2$: Distribution studies and dose calculated for $^{193m}Pt$ and $^{195m}Pt$. *J. Nucl. Med.*, **14**, 191.

MILLAR, B.C. (1982). Hypoxic cell radiosensitizers as potential adjuvants to conventional chemotherapy for the treatment of cancer. *Biochem. Pharmacol.*, **31**, 2439.

NIAS, A.H.W. & SZUNIEL, I.I. (1977). The effects of *cis*-dichlorobiscyclopentylamineplatinum(II) PAD and *cis*-dichlorobisisopropylamine trans-hydroxy platinum(IV) CHIP and radiation and CHO cells. *J. Clin. Hematol. Oncol.*, **7**, 562.

OVERGAARD, J. & KHAN, A.R. (1981). Selective enhancement of radiation response in a C3H mammary carcinoma by *cis*-platin. *Cancer Treat. Rep.*, **65**, 501.

RAUTH, A.M. MOHINDRA, J.K. & TANNOCK, I.F. (1983). Activity of mitomycin C for aerobic and hypoxic cells *in vitro* and *in vivo*. *Cancer Res.*, **43**, 4154.

ROCKWELL, S. (1977). *In vivo-in vitro* tumour systems: New models for studying the response of tumors to therapy. *Lab. Animal Sci.*, **27**, 831.

ROCKWELL, S. (1978). Cytotoxic and radiosensitizing effects of hypoxic cell sensitizers on EMT6 mouse mammary tumor cells *in vivo* and *in vitro*. *Br. J. Cancer*, **37** (Supp. III) 212.

ROCKWELL, S. (1982). Cytotoxicities of mitomycin C and X-rays to aerobic and hypoxic cells *in vitro*. *Int. J. Radiat. Oncol. Biol. Phys.*, **8**, 1035.

ROCKWELL, S. & KALLMAN, R.F. (1973). Cellular radiosensitivity and tumor radiation response on the EMT6 tumor cell system. *Radiat. Res.*, **53**, 281.

ROCKWELL, S.C., KALLMAN, R.F. & FAJARDO, L.F. (1972). Characteristics of serially transplanted mouse mammary tumor and its tissue-culture-adapted derivative. *J. Natl Cancer Inst.*, **49**, 735.

ROCKWELL, S. & KENNEDY, K.A. (1979). Combination therapy with radiation and mitomycin C: Preliminary results with EMT6 tumor cells *in vitro* and *in vivo*. *Int. J. Radiat. Oncol. Biol. Phys.*, **5**, 1673.

ROCKWELL, S., MROCZKOWSKI, Z., & RUPP, W.D. (1982). Evaluation of 2-amino-5-nitrothiazole as a hypoxic cell radiosensitizer. *Radiat. Res.*, **90**, 575.

ROSE, C.M., MILLER, J.L., PEACOCK, J.H., PHELPS, T.A. & STEPHENS, T.C. (1980). Differential enhancement of melphalan cytotoxicity in tumor and normal tissue by misonidazole. In: *Radiation Sensitizers: Their Use in the Clinical Management of Cancer*, Brady, L.W. (ed) p. 250. Masson Publishing: New York.

ROSE, W.C., SCHURIG, J.E., HUFTALEN, J.B. & BRADNER, W.T. (1982). Antitumor activity and toxicity of *cis*-platin analogs. *Cancer Treat. Rep.*, **66**, 135.

SCHABEL, F.M., TRADEU, M.W., LASTER, W.R., Jr., CORBETT, T.H. & GRISWOLD, D.P. Jr., (1979). *Cis*-dichlorodiammineplatinum(II): Combination chemotherapy and cross-resistance studies with tumors of mice. *Cancer Treat. Rep.*, **63**, 1459.

SIEMAN, D.W. (1982). Potentiation of chemotherapy by hypoxic cell radiation sensitizes – a review. *Int. J. Radiation Oncol. Biol. Phys.*, **8**, 1029.

STEEL, G.G. & PECKHAM, M.J. (1979). Exploitable mechanisms in combined radiotherapy-chemotherapy: The concept of additivity. *Int. J. Radiat. Oncol. Biol. Phys.*, **5**, 85.

TEICHER, B.A., LAZO, J.S. & SARTORELLI, A.C. (1981). Classification of antineoplastic agents by their selective toxicities toward oxygenated and hypoxic tumor cells. *Cancer Res.*, **41**, 73.

TEICHER, B.A. & SARTORELLI, A.C. (1980). Nitrobenzyl halides and carbamates as prototype bioreductive alkylating agents. *J. Med. Chem.*, **23**, 955.

TEICHER, B.A. & SARTORELLI, A.C. (1981). Selective attack of hypoxic tumor cells. In: *Design of Models for Screening of Therapeutic Agents for Cancer*, Fidler, I.J. & White, R.J. (eds) p. 19. Van Nostrand Reinhold, New York.

VESELY, J. (1982). Synergistic effect of *cis*-dichlorodiammineplatinum and 5-aza-2′-deoxycytidine on mouse leukemic cells *in vivo* and *in vitro*. *Int. J. Cancer*, **29**, 81.